# Using pedigree reconstruction to estimate population size: genotypes are more than individually unique marks

**DOI:** 10.1002/ece3.538

**Published:** 2013-04-08

**Authors:** Scott Creel, Elias Rosenblatt

**Affiliations:** 1 Department of Ecology, Montana State UniversityBozeman, Montana, 59717; 2 Zambian Carnivore ProgrammeBox 80, Mfuwe, Eastern Province, Zambia

**Keywords:** Census, lion, mark-recapture, pedigree reconstruction, population estimate, population size

## Abstract

Estimates of population size are critical for conservation and management, but accurate estimates are difficult to obtain for many species. Noninvasive genetic methods are increasingly used to estimate population size, particularly in elusive species such as large carnivores, which are difficult to count by most other methods. In most such studies, genotypes are treated simply as unique individual identifiers. Here, we develop a new estimator of population size based on pedigree reconstruction. The estimator accounts for individuals that were directly sampled, individuals that were not sampled but whose genotype could be inferred by pedigree reconstruction, and individuals that were not detected by either of these methods. Monte Carlo simulations show that the population estimate is unbiased and precise if sampling is of sufficient intensity and duration. Simulations also identified sampling conditions that can cause the method to overestimate or underestimate true population size; we present and discuss methods to correct these potential biases. The method detected 2–21% more individuals than were directly sampled across a broad range of simulated sampling schemes. Genotypes are more than unique identifiers, and the information about relationships in a set of genotypes can improve estimates of population size.

## Introduction

Conservation and management of wildlife populations require information on population size, but this is usually difficult to obtain for species that are rare or elusive. Many endangered species (exemplified by large carnivores) are both rare and elusive, and accurate estimates of total population size for such species are not common. Methods have been developed to extract DNA and determine microsatellite genotypes from hair (Goossens et al. 1998; Flagstad et al. 1999; Woods et al. 1999; Sloane et al. 2000), feces (Taberlet et al. 1996, 1999; Gagneux et al. 1997; Kohn and Wayne 1997; Kohn et al. 1999) and less direct sources of cells (Parsons et al. 1999; Valiere and Taberlet 2000). Because hair and fecal samples can be collected without capturing or handling the animal, these methods have great promise for population estimation. Genotypes can be used to estimate population size in several ways. Most directly, the number of genotypes is an estimate of the minimum population size, which can be identified by the asymptote of a curve relating the number of distinct genotypes to the number of samples (Kohn et al. 1999) or by rarefaction (Kalinowski 2005). Capture-mark-recapture (CMR) methods of estimating population size can be applied to genetic data if individuals are sampled sufficiently often to estimate capture probabilities (Otis et al. 1978; Seber 1982, 1986; Boulanger et al. 2004; Kendall et al. 2009). Genetic CMR methods all rely on the logical argument that population size 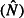 can be estimated by the number of genotypes (or “captures” *C*) divided by the probability of capture 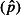. With repeated sampling 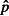 can be estimated directly from individual capture histories (Otis et al. 1978). These direct genetic census methods have been applied to several large carnivore species including brown bears (*Ursus arctos*: Taberlet et al. 1999; Boulanger et al. 2004; Kendall et al. 2009), wolves (*Canis lupus*: Creel et al. 2003), mountain lions (*Panthera concolor*: Ernest et al. 2000; Sawaya et al. 2011), and coyotes (*Canis latrans*: Kohn et al. 1999). Direct genetic census methods are virtually identical to other CMR methods in their underlying logic; genotypes simply substitute for any other type of mark that allows individuals to be identified.

The use of genotypes as individual identifiers does not necessarily eliminate bias in estimates of population size (and survival rates) due to “misread” marks. If the number or variability in genetic markers is low, two individuals may have the same genotype. In this case, the number of unique captures (*C*) is underestimated and the probability of capture 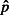 is overestimated leading to underestimation of population size 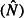 (Mills et al. 2001). This “shadow effect” can be corrected by the use of additional genetic markers or markers with greater variation among individuals. If individuals are sampled repeatedly and genotyping errors occur (allelic dropout and misprinting are relatively common with noninvasive sample types that have low DNA yield and poor preservation), then (*C*) is overestimated and 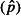 is underestimated leading to overestimation of 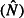. This “ghost effect” (Creel et al. 2003) can be corrected by procedures to check for and eliminate genotyping errors (Taberlet et al. 1996; Taberlet et al. 1999) or by using methods that allow for imperfect genotyping (Creel et al. 2003; Kalinowski et al. 2006). Ironically, the ghost effect becomes stronger if the number of loci is increased to eliminate the shadow effect, or if the number of samples is increased to better estimate 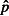 (Creel et al. 2003). Finally, it can be difficult to identify distinct sampling occasions for population estimates using genetic CMR if noninvasive samples (such as hair) can persist in the environment for long periods. As with CMR studies using other types of marks, population estimates using genetic CMR often have wide confidence limits because recapture probabilities are low (Mills et al. 2001; Lukacs and Burnham 2005).

Indirect genetic census methods based on pedigree reconstruction have also been developed (Jones and Avise 1997a,b; Pearse et al. 2001; Israel and May 2010). For example, in a study of painted turtles (*Chrsemys picta*), the set of genotypes “captured” was extended to include individuals who were never directly sampled, but whose genotype could be inferred by pedigree reconstruction (Pearse et al. 2001). This study took advantage of the opportunity to sample many offspring in a single nest. With multi-locus microsatellite genotypes from many offspring and one parent (the female attending the nest), the likely genotype of an un-sampled parent (in this case, the father) could be inferred with confidence. The resulting set of genotypes was then analyzed with the normal CMR logic to estimate population size. The inclusion of individuals inferred to be present from pedigree reconstruction improved the population estimate substantially, compared to estimates based on direct CMR analysis (Pearse et al. 2001).

Pedigree reconstruction can detect the genetic fingerprint (and thus the presence) of un-sampled individuals. This distinguishes pedigree reconstruction from typical direct genetic census methods, and presents a potential alternative method to estimate population size. This alternative may be more powerful and precise, because it does not reduce the information in a multi-locus genotype into a “mark” that simply identifies an individual in the same manner that a colored leg band or ear tag would identify it. In addition to serving as individually unique identifiers, genotypes contain information about population structure (genetic relationships among individuals), and that information can be used to improve estimates of population sizes and trends (Luikart et al. 2010; Tallmon et al. 2010).

Several excellent recent reviews discuss the range of genetic markers suitable for pedigree reconstruction, and appropriate methods of analysis (Blouin 2003; Morin et al. 2004; Anderson and Garza 2006; Kalinowski et al. 2006; Wagner et al. 2006; Koch et al. 2008; Pemberton 2008; Wang and Santure 2009; Jones et al. 2010; Riester et al. 2010). For the purposes of this article, we restrict our discussion to comparing of the genotypes of a set of offspring to the genotype of one parent and thus inferring the genotype of the other parent, even though it was not directly sampled. Some methods of pedigree reconstruction can provide insight into secondary relationships between individuals (and thus can potentially be used to infer the existence of un-sampled individuals across generational gaps or past first-order relationships), but we leave this for later.

**Figure 1:**
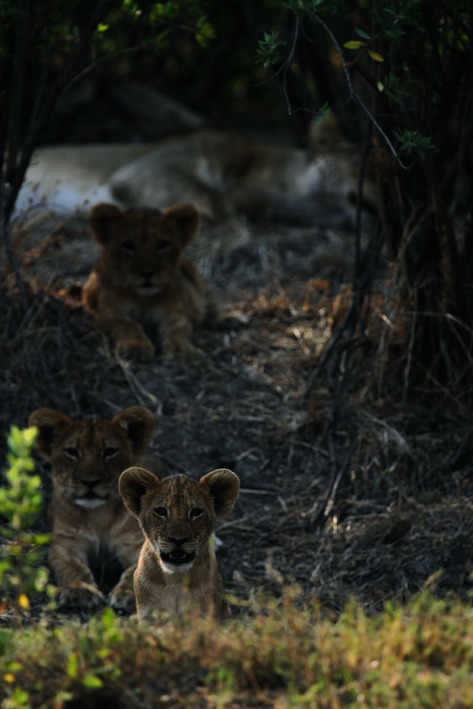
Lion cubs with their resting mother in South Luangwa National Park, Zambia. Photo by E. Rosenblatt.

Until now, pedigree reconstruction methods have been used to estimate the number of breeding individuals in a population, rather than total population size (Jones and Avise 1997a,b; Nielsen et al. 2001; Pearse et al. 2001; Koch et al. 2008; Israel and May 2010). Because an individual must breed in order to appear in a pedigree, this constraint initially seems unavoidable. Here, we use a simulation model to show that pedigree reconstruction can be used to estimate total population size. From the perspectives of conservation and management, total population size is often of greater interest than the number of breeders (for example, to evaluate the effect of human harvest on population dynamics; Cooley et al. 2009; Creel and Rotella 2010; Packer et al. 2010). We present formulas to estimate the size of a population by estimating the numbers of both breeding and nonbreeding adults, and use Monte Carlo simulation to evaluate the bias and precision of the estimates. The simulated population has demographic properties derived from African lions in Zambia (*Panthera leo*; Fig. 1; Becker et al. 2013a) so that the method is tested for a scenario that exemplifies a species of conservation and management concern. Finally, we use the simulation to explore the effects of variation in sampling methods and sampling intensity on the bias and precision population estimates.

## Using Pedigree Reconstruction to Estimate Population Size Including Individuals that did not Breed

We can estimate total population size 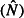 as the sum of individuals directly sampled (*N*_s_), individuals that bred and whose presence was inferred by pedigree reconstruction (*N*_in_), and individuals who did not breed and therefore remained “invisible” to pedigree reconstruction (*N*_iv_).


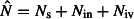
(1)

where


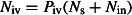
(2)


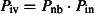
(3)


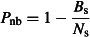
(4)

and


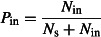
(5)

The variables are as follows:

*N*_s_ = number of individuals sampled

*N*_in_ = number of individuals inferred by pedigree reconstruction

*N*_iv_ = number of individuals invisible to pedigree reconstruction (unsampled nonbreeders)

*P*_iv_ = probability of an individual being “invisible”

*P*_nb_ = probability of an individual not breeding

*P*_in_ = probability of inferring an individual's presence by pedigree reconstruction

*B*_s_ = number of breeders sampled

The number of individuals directly sampled and genotyped (*N*_s_) requires no special explanation. *N*_in_ is the number of individuals that were not directly sampled, but whose presence could be inferred by pedigree reconstruction because they bred with sampled mates and left offspring that were also sampled. Logically, the number of “invisible” individuals (*N*_iv_) that were not detected by sampling or by pedigree reconstruction can be estimated as the proportion of individuals that were invisible to the pedigree (*P*_iv_, animals that neither bred nor were directly sampled) multiplied by the total size of the pedigree (*N*_s_ + *N*_in_) (eq. 2). Under any given sampling scheme, the proportion of individuals invisible to both direct sampling and pedigree reconstruction (*P*_iv_) is simply the product of two known quantities: the proportion of detected individuals that were detected only by pedigree reconstruction and not by direct sampling (*P*_in_) and the proportion of individuals that did not breed, and thus could not be inferred by pedigree reconstruction (*P*_nb_) (eq. 3). Equation 3 assumes that the probability of obtaining a sample from an individual is independent of its breeding status, which is likely to be correct for many methods of sampling. Both these probabilities can be estimated by the data. The probability of breeding (and thus the probability of not breeding, *P*_nb_) can be estimated from the number of directly sampled individuals (*N*_s_) and the subset of these that were known to breed (*B*_s_) because they had descendants in the pedigree (eq. 4). The probability of being detected only by reconstruction (and not by a direct sample) is also easily estimated from the pedigree itself (eq. 5). Equations (1–5) simplify by substitution as shown below:


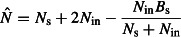
(6)

This estimator uses the information in a set of genotypes to estimate population size as the sum of three segments of a population: (1) the number of individuals directly sampled, (2) the number of individuals who were not sampled, but whose existence could be directly inferred by pedigree reconstruction, and (3) the number of individuals that were not sampled and whose existence could not have been inferred by pedigree reconstruction, because they left no offspring. The logic of this estimator is similar in one way to the logic of CMR estimators, because the number of individuals “captured” (*N*_s_ here, *C* in the context of CMR) is adjusted upward to account for individuals that were not captured. The logic differs because this process of adjustment does not simply treat genotypes as individual identifiers, but instead takes advantage of the information that genotypes provide about population structure. Below, we use simulation to confirm that the estimator is unbiased and precise with reasonable sampling effort.

## A Caveat About Demographic Closure

This method estimates the size of the adult population within the sampling period. The method (as presented above) does not account for the possibility that a pedigree can contain a set of individuals that were not all alive at the same time. For population surveys based only on direct genotypes, noninvasive genetic samples can usually be collected intensively within a period that has been tested for population closure by species-specific mark-recapture surveys (i.e., 60 days for snow leopards (*Uncia uncial*; Jackson et al. 2005), 59 days for jaguars (*Panthera onca*; Silver et al. 2004), and 250 days for leopards and tigers (*Panthera pardus fusca* and *Panthera tigris tigris*; Wang and Macdonald 2009). However, it is advisable for users of this method to test and confirm population closure using one of multiple software packages available, or using direct data on the rates (and timing) of mortality, reproduction, immigration, and emigration. We return to this issue in the results and discussion, because the assumption of demographic closure is more likely to be violated when the set of genotypes includes inferred parents (which can be dead by the time that their existence is inferred).

## Simulations to Evaluate Bias and Precision of the New Population Size Estimator

We evaluated the population size estimator (eq. 6) by simulation, sampling from a modeled lion population that we created using demographic data from a lion population in Eastern Zambia's Luangwa Valley (Becker et al. 2013a). By creating a simulated population and then sampling from it stochastically, we could do the following: (1) Compare the population estimate to the true population size across a range of realistic sampling schemes (e.g., including or excluding samples from juveniles) and sampling intensities (ranging from 10% to 90% of the population that was not sampled in previous years), and (2) test the inferential gains from including inferred and “invisible” individuals in the estimate. We based the simulated population on demographic data from lions because they typify large, wide-ranging endangered carnivores, for which there are few logistically tractable methods that provide precise estimates of population size. We used data from Luangwa Valley lions to parameterize the model, because this population is now being sampled for an empirical test of the method, and because it is affected by two conservation issues of importance for the species (and for large carnivores in general): harvest by trophy hunters (Creel and Creel 1997; Whitman et al. 2007; Packer et al. 2009; Creel and Rotella 2010) and mortality and prey depletion due to illegal harvest, in this case, wire snaring (Becker et al. 2013a,b).

We emphasize that the model was designed to test the population size estimator with a simulated population of known size, with known parent–offspring relationships. The intention of the simulation was not to make detailed inferences about the dynamics of a specific lion population. Using MS Excel, we created an individual-based model with a binomially-distributed initial adult sex ratio with a mean of 0.92 (proportion females; the Luangwa Valley lion population used to parameterize the model is male-depleted as a consequence of trophy hunting), a binomially-distributed cub sex ratio with a mean of 0.50, and an initial age-distribution matching that observed in the Luangwa population. The initial distribution of individuals across age classes 0–8 years was 0.10, 0.17, 0.17, 0.18, 0.12, 0.07, 0.08, 0.07, and 0.05 (Becker et al. 2013a). Age-specific mean survival rates from age classes 0–12 were 0.63, 0.91, 0.93, 0.95, 0.95, 0.94, 0.91, 0.82, 0.46, 0.26, 0.18, 0.10, and 0.05, (Becker et al. 2013a) and survival for each age class was assumed to be a binomial process (i.e., we did not model extra-binomial variation in survival rates within age classes). Becker et al. (2013a) reported fecundity as number of cubs per female, but our simulation tracked newborns by drawing from a normal distribution of litter sizes, and then assigning the value drawn to each female that reproduced (i.e., reproduction was modeled as a binomial process qualitatively matching that was observed in Luangwa lionesses: see below). The assignment of stochastic litter sizes used Schaller's (1972) mean litter size of 2.4 (±0.5 SD) cubs per litter, because we lacked sufficient data from Luangwa.

We assumed that females began reproducing at 4 years of age and that once a female reproduced, she did not reproduce for 2 years regardless of the fate of her cubs. The composition of mating pairs was assigned randomly among adults alive in that year. A useful refinement of this model for a more species-specific application would be to examine the effect of population subdivision and mating within and among prides (Gilbert et al. 1991; Packer et al. 1991; Spong et al. 2002). The initial population size was 100 individuals, and the individual-based model was run for a period of 15 years. Individuals were tracked throughout their lifespan to identify parent-offspring relationships. The first 10 years were used as burn-in, and years 11–15 were used to simulate the collection of genotypes that we then used to estimate population size with equation 6.

**Figure 2:**
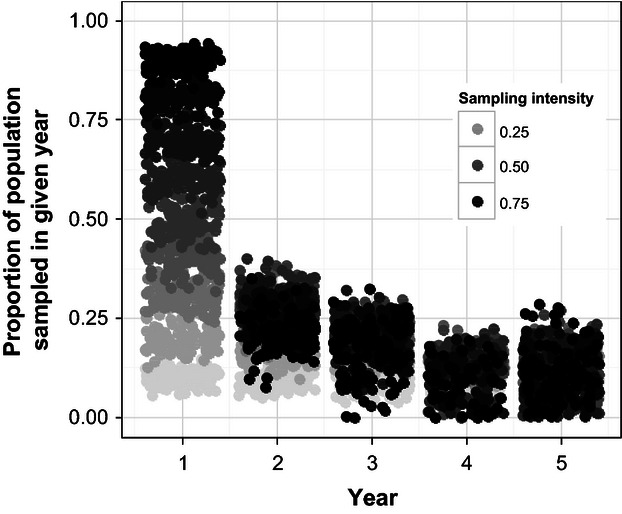
The intensity of sampling required to obtain samples from a fixed proportion of the population declines through time, in a scenario with realistic turnover of individuals within a population. The results from 4500 Monte Carlo simulations of sampling 10–90% of the individuals in a lion population showed, as expected, that this decrease was most pronounced for the most intensive sampling. The proportion of the population that must be sampled in a given year to maintain the desired sampling coverage typically dropped to ≤20% within 3 years. These results are from simulated sampling that included juveniles, which increased the rate of turnover and thus increased the required sampling.

Varying the intensity of sampling from 10% to 90% (in increments of 10%) of the previously unsampled portion of the population each year, we ran 100 replicates of the simulation for each sampling intensity level for each of two sampling schemes. As sampled individuals accumulate in a population, the annual sampling effort required to maintain constant sampling coverage declines. Figure 2 shows the sampling intensity that was required in each year to maintain sampling coverage of 10–90% of the lion population in 9000 iterations of our Monte Carlo model. The details of this pattern are expected to vary in a manner that depends on the rate of individual turnover in a population. In general, long generation times and low population growth rates (both typical of large carnivores) will allow sampling effort to asymptote at a lower value for a given level of coverage.

We modeled two sampling schemes. One sampling scheme mimicked biopsy darting (Karesh et al. 1987), which provides high quality tissue samples for extraction of DNA, but it cannot be used with juveniles of many species. For the purposes of this model, individuals <2 years old were excluded from sampling. The other sampling scheme mimicked the collection of fecal samples, a technique with theoretically no age limitation (though the yield and quality of DNA is lower and very young lions [<3–4 weeks] may be difficult to sample due to lower visibility). We tracked the cumulative number of sampled, inferred and “invisible” individuals, which of these individuals were still alive in each year, the population size in each year, the number of sampled breeding individuals, and 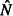 (following eq. 6). We inferred the existence of an un-sampled individual if at least one of its offspring and the other parent were sampled; that is we assumed that genotypes were sufficiently powerful to infer parents without error. This assumption is likely to hold for pedigree analysis based on a large number of single-nucleotide polymorphisms (SNPs) (e.g., genotyping by sequencing can now yield 100,000s of SNPs per genotyped individual, and species-specific SNP chips can efficiently provide 96 or 384 SNPs). Although it is conceptually possible to extend the method using inferences about second-order genetic relationships with such data, we did not address this possibility.

## Results and Discussion

**Figure 3:**
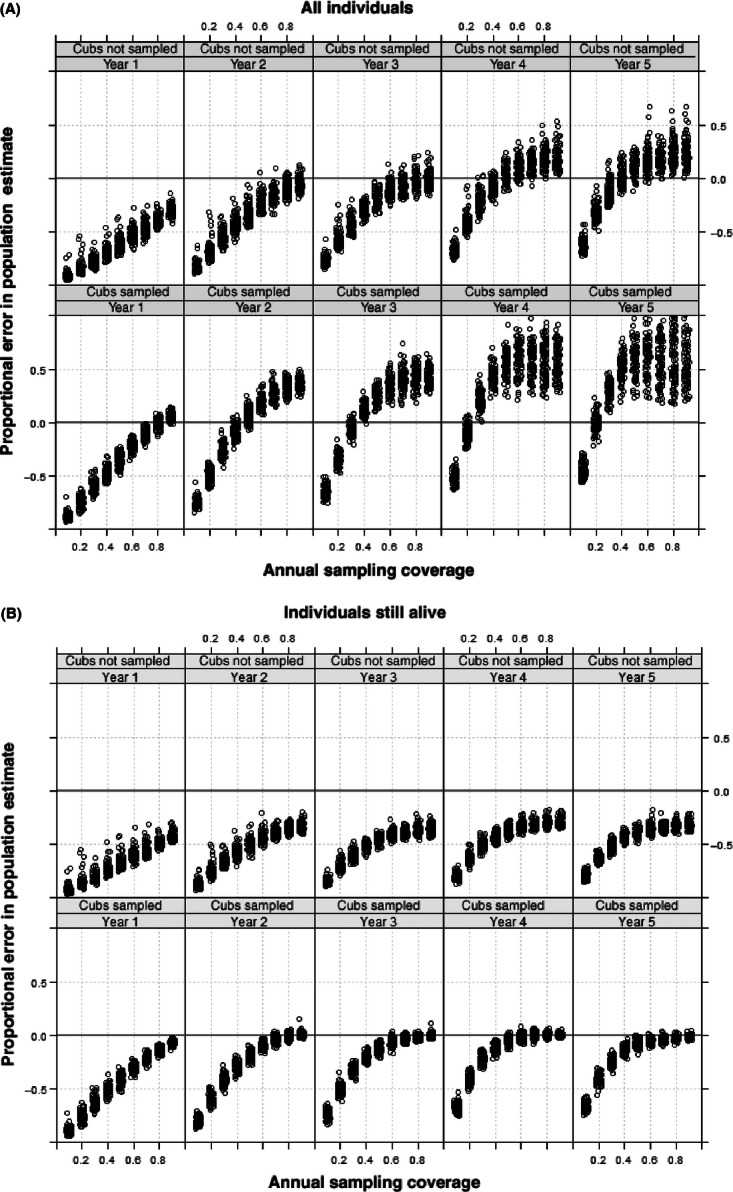
Results of 9000 Monte Carlo simulations comparing population estimates from pedigree reconstruction (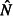 from equation 6) to the true population size of the simulated lion population described in the text. In each panel, the ordinate shows the difference between estimated population size and true population size, as a proportion of true population size, so that zero represents an unbiased estimate, negative values indicate underestimation, and positive values indicate overestimation. In each panel, the abscissa shows the proportion of the population sampled annually. The five panels in each row show changes across five consecutive years of sampling, with estimates from 1 year of sampling on the left, and 5 years of sampling on the right. The bottom row shows results from simulations in which juveniles were sampled, and the top row shows results from simulations in which juveniles were not sampled. (A) In this implementation, the population estimator includes individuals that died after being sampled. With sampling over a long period of sampling, population size is often overestimated (particularly with high sampling coverage) because the population estimate includes individuals that were not alive at the end of the sampling interval. (B) In this implementation, the population estimator excludes individuals that were sampled or inferred to exist, but died prior to the year for which the population estimate is produced. With adequate sampling, the population estimator is unbiased and precise.

Comparing estimated population size 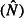 to true population size across 9000 simulations with a broad range of sampling scenarios confirms that the estimator is fundamentally unbiased. The population estimate converges on the true population size with adequate duration and intensity of sampling (Fig. 3B, lower right panels). The method can also provide precise estimates, particularly in comparison with many of the methods used to estimate population size in difficult-to-count species like large carnivores (Fig. 3B lower right panels). However, two issues related to sampling can cause the population estimate to be biased.

First, the method can potentially overestimate population size if the pedigree includes individuals that were sampled, or whose presence was inferred by reconstruction, over a long period (Fig. 3A, particularly lower right panels). This problem is not unique to this method (Pollock et al. 1990). For any method that relies on data aggregated over an appreciable interval, the population estimate might include individuals that died before the end of the period. Pedigree reconstruction tends to amplify this basic problem, because an individual can be inferred by pedigree reconstruction after it is already dead. If one has an estimate of the annual rate of mortality, then it is conceptually simple to remove this overestimation bias by accounting for mortality during the sampling period. Following equation 7, the population estimate at the end of the period is a weighted sum of the number of individuals first detected in each year, with weights equal to the probability of survival (*s*) from the year of detection to the end of the entire sampling period. The annual *N*_i_ values in this summation should include only the first (or last) detection of each individual, to avoid double counting.


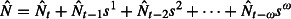
(7)

Equation 7 reduces to yield equation 8,


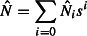
(8)

where *i* is an index of years (or other intervals with equal lengths) prior to the final sample. One could also reduce or eliminate this overestimation problem by sampling intensively over an interval short enough for little mortality to occur, though obtaining adequate samples in a short period may not be tractable for many species (Figs. 3). In this situation, one might treat as a count rather than a final estimate of population size, and use well-established CMR methods to produce the final estimate (Pollock et al. 1990; Kendall 1999; Lukacs and Burnham 2005).

Second, the type, duration, and intensity of sampling must be adequate, or population size will be underestimated. Not surprisingly, the method works better if juveniles can be sampled, because this increases the likelihood of inferring the existence of parents that were not directly sampled. Sampling for a period of 2 years (with juveniles sampled), the population estimate is typically within 10% of true population size if ≥50% of the population is sampled. As the duration of sampling increases, the sampling intensity required to maintain this level of accuracy decreases, but not by much. Thus, the method is best suited to species and contexts in which it is reasonable to expect that ∼40% of previously unsampled individuals can be sampled if 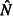 is to be considered a direct estimate of population size. For species and contexts in which this intensity of sampling is not likely to be possible, one could also avoid underestimation by treating 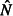 from equation 6 as a count, rather than a population estimate (Pollock et al. 1990; Kendall 1999). If we consider 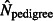 to be a count (rather than an estimate of population size) and apply standard CMR logic, then 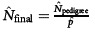, where 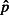 is an estimate of the proportion of the population included in the pedigree. This might be estimated simply using the logic of the Lincoln–Peterson method or its extensions (Seber 1973; Williams et al. 2002). Finally, one might adjust the count to produce a final estimate of population size by estimating the asymptote of an accumulation curve or rarefaction analysis, as is sometimes done with simple counts of genotypes (Kohn et al. 1999; Gotelli and Colwell 2001).

**Figure 4:**
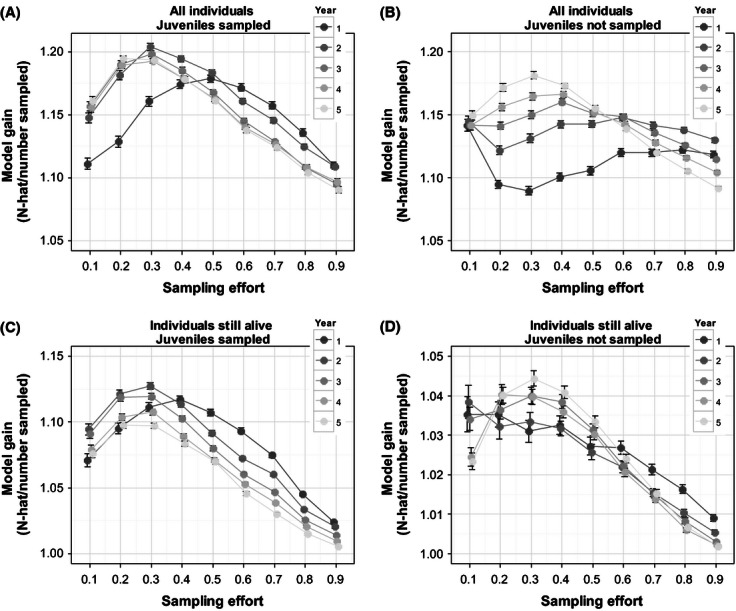
Gains in the detection of unsampled individuals through the use of equation 6, relative to a count of directly sampled individuals. The ordinate plots the increase in estimated population size, relative to the number of individuals detected by direct sampling, as functions of sampling effort (from 10% to 90% of the population sampled), years of sampling (from 1 to 5), and sampling type (juveniles included or excluded). (A, B) Estimates of population size include individuals that died prior to the year of the estimate. (C, D) Estimates of population size exclude individuals that died prior to the year of the estimate.

Underestimation and overestimation biases can fortuitously negate one another (e.g., Fig. 3A, upper middle panel for 3 years of sampling with cubs not sampled), but this outcome should not be mistaken for validation of the method. The method reliably provided unbiased estimates of population size only when juveniles were sampled, sampling intensity exceeded 40% of the population and extended over several years, and individuals that had died were excluded from the estimate. These limitations are important, but the simulations also confirm the basic premise that pedigree reconstruction can provide an unbiased and precise estimate of population size, by taking advantage of the information about population structure that is contained within genotypes. Figure 4 illustrates the way in which this information increased the estimate of population size, relative to a simple count of the individuals sampled. Under the sampling scenarios we considered, pedigree reconstruction increased the number of individuals detected by 2–20%. In general, the biggest gains occurred when 30–40% of the population was sampled, including juveniles. Sampling juveniles is important to take advantage of the method because this increases the likelihood of inferring the presence of an un-sampled parent. At very low sampling intensities, it is unlikely that an offspring and one of its parents will be sampled, so little power is gained over simply counting unique genotypes. At high sampling intensities, it is likely that any individuals that could be inferred would also be directly sampled.

Given the strong effect of age-restricted sampling methods in our simulations, it is important that field studies carefully consider the method of obtaining DNA. The ideal use of this method would be in a study that intensively collected noninvasive (fecal or hair) and/or nondestructive (tissue or blood) samples intensively across a large area with the intent of collecting samples from as many individuals as possible. Using multiple sample types would increase sampling efficiency and reduce the possibility of sampling that was biased by age or sex.

The ideal genetic marker for any pedigree reconstruction would provide high resolution to provide reliable relatedness estimates beyond first-order relationships, thereby facilitating pedigree reconstruction. There is an emerging preference for SNPs as a low-cost, stable marker that can provide suitable genetic resolution (Smouse 2010). Although microsatellites require one half to one quarter as many markers to provide equivalent information content, SNPs provide stable, plentiful markers, the effect of mutation on these markers is predictable by simple mutation models, genotyping errors are minimal, and markers are present even in severely damaged genetic samples (Morin et al. 2004; Anderson and Garza 2006; Jones et al. 2010; Mesnick et al. 2011). We anticipate that the method outlined in this article will be applied with SNPs, perhaps in the context of genotyping by sequencing, which can provide more than 100,000 SNPs per genotyped individual.

In summary, genotypes uniquely identify individuals, but they provide information beyond identity that can be used to estimate population size. In simulations that assumed that genotypes would provide enough information to identify an un-sampled parent when the other parent and at least one offspring was sampled, pedigree reconstruction increased the count of sampled individuals by 2–20% depending on sampling methods. This constitutes a valuable increase in the power to detect individuals with no extra sampling, relative to methods that simply treat genotypes as unique identifiers. Particularly if combined with CMR methods of population estimation, pedigree reconstruction offers a promising method of increasing the power of noninvasive genetic methods to estimate population size.
